# Low-frequency electrical stimulation of bilateral hind legs by belt electrodes is effective for preventing denervation-induced atrophies in multiple skeletal muscle groups in rats

**DOI:** 10.1038/s41598-022-25359-z

**Published:** 2022-12-08

**Authors:** Hiroyuki Uno, Shohei Kamiya, Ryuji Akimoto, Katsu Hosoki, Shunta Tadano, Karina Kouzaki, Yuki Tamura, Takaya Kotani, Mako Isemura, Koichi Nakazato

**Affiliations:** 1 HOMER ION Co., Ltd., Shinsen 17-2, Shibuya-ku, Tokyo, 150-0045 Japan; 2 School of Health and Sport Science, Nippon Sport Science University, 7-1-1 Fukazawa, Setagaya-ku, Tokyo, 158-8508 Japan

**Keywords:** Biochemistry, Physiology

## Abstract

Belt electrode skeletal muscle electrical stimulation (B-SES) can simultaneously contract multiple muscle groups. Although the beneficial effects of B-SES in clinical situations have been elucidated, its molecular mechanism remains unknown. In this study, we developed a novel rodent B-SES ankle stimulation system to test whether low-frequency stimulation prevents denervation-induced muscle atrophy. Electrical stimulations (7‒8 Hz, 30 min) with ankle belt electrodes were applied to Sprague–Dawley rats daily for one week. All animals were assigned to the control (CONT), denervation-induced atrophy (DEN), and DEN + electrical stimulation (ES) groups. The tibialis anterior (TA) and gastrocnemius (GAS) muscles were used to examine the effect of ES treatment. After seven daily sessions of continuous stimulation, muscle wet weight (n = 8–11), and muscle fiber cross-sectional area (CSA, n = 4–6) of TA and GAS muscles were lower in DEN and DEN + ES than in CON. However, it was significantly higher in DEN than DEN + ES, showing that ES partially prevented muscle atrophy. PGC-1α, COX-IV, and citrate synthase activities (n = 6) were significantly higher in DEN + ES than in DEN. The mRNA levels of muscle proteolytic molecules, Atrogin-1 and Murf1, were significantly higher in DEN than in CONT, while B-SES significantly suppressed their expression (p < 0.05). In conclusion, low-frequency electrical stimulation of the bilateral ankles using belt electrodes (but not the pad electrodes) is effective in preventing denervation-induced atrophy in multiple muscles, which has not been observed with pad electrodes. Maintaining the mitochondrial quantity and enzyme activity by low-frequency electrical stimulation is key to suppressing muscle protein degradation.

## Introduction

Inactivity, such as after surgery or in the elderly, causes skeletal muscle atrophy and weakness, impairing daily activity. Prevention of skeletal muscle wasting is critical for extending healthy life expectancy and maintaining the quality of life. In age-related skeletal muscle atrophy, sarcopenia, denaturing of the neuromuscular junction is a key event that induces atrophy^1^. Therefore, the prevention of denervation-induced atrophy is important for rescuing skeletal muscle atrophy.

Mitochondria are one of the causes of muscle atrophy associated with inactivity, and it has been shown that the number and activity of mitochondria in skeletal muscle are reduced by inactivity^2,3^. It has been suggested that an increase in reactive oxygen species (ROS) produced by mitochondrial abnormalities leads to increased muscle protein degradation^4^ and increased matrix metalloproteinase due to changes in intracellular calcium ion concentrations leading to extracellular matrix degradation^5^, resulting in progressive muscle atrophy. It has been reported that when denervation induces muscle atrophy in experimental animals, PGC-1α activity, which is involved in mitochondrial biosynthesis, COXIV protein, which reflects mitochondrial mass^2,3^, and citrate synthase (CS) are decreased, as well as a quantitative decrease in mitochondrial enzyme activity^6^.

Muscle atrophy is partly suppressed by inducing muscle contraction via electrical muscle stimulation (EMS) using pad electrodes^7^. Low-frequency EMS-induced muscle contraction, such as twitch contraction, increases mitochondrial volume and enzyme activity and suppresses muscle proteolytic signaling, which is enhanced during muscle atrophy^8,9^. This suggests that the maintenance of mitochondrial volume and enzyme activity and suppression of the accompanying increase in muscle proteolytic signaling are important for minimizing muscle atrophy with EMS. However, it is difficult to exercise multiple muscle groups simultaneously with pad electrode electrical stimulation because the electrodes are usually placed directly above the target muscle and the current is only applied to target a single muscle^7^.

Belt electrode skeletal muscle electrical stimulation (B-SES), unlike electrical stimulation with pad electrodes, is an electrical stimulation method that can stimulate and drive multiple muscle groups simultaneously via electrical stimulation of the entire lower limb in a tubular fashion using belt-shaped electrodes. Clinical studies have reported that B-SES simultaneously activates multiple muscle groups, suppresses muscle atrophy, and improves motor function in critically ill patients^10–12^. Although the beneficial effects of B-SES have been clinically studied, the underlying molecular mechanisms remain unknown.

In this study, we first developed a rodent B-SES model and tested whether electrical stimulation could be successfully applied to both legs between two belt electrodes and activated multiple muscle groups by assessing glycogen consumption and AMP-activated protein kinase (AMPK) phosphorylation. Furthermore, due to a decrease in neurotransmission from motor nerves to skeletal muscles with aging^1^, we evaluated whether chronic electrical stimulation with B-SES prevent muscle protein degradation and muscle atrophy and increase mitochondrial biosynthesis, muscle mass and enzyme activity in the sciatic nerve denervation model. Finally, we placed pad electrodes in the same position as the belt electrodes (behind both ankle joints) with the same current per area and compared the effects of chronic electrical stimulation on muscle atrophy suppression with B-SES treatment.

## Results

### Glycogen content and AMPK phosphorylation after acute belt electrode skeletal muscle electrical stimulation

**Figure 1:**
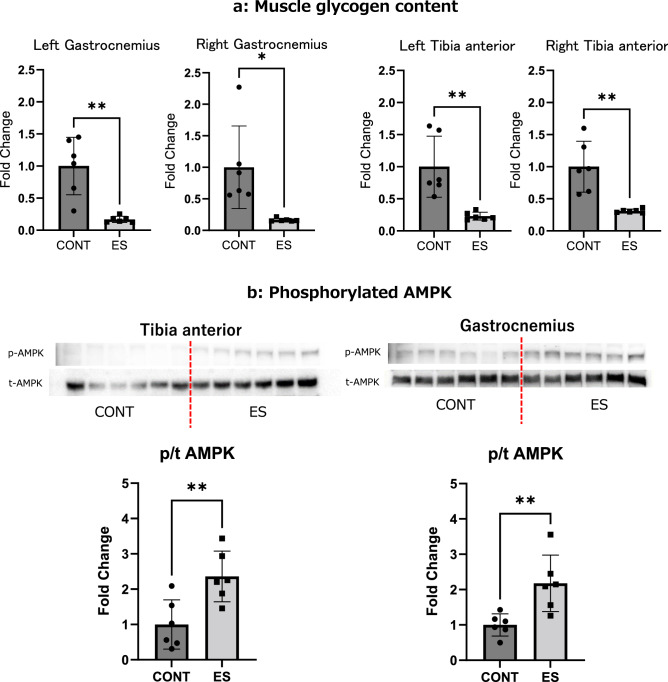
Glycogen content (**a**) and phosphorylated AMPK (**b**) of TA and GAS after belt electrode acute stimulation. (**a**) Glycogen content of GAS and TA (**b**) Phosphorylated AMPK of GAS and TA. Mean ± SD. An unpaired *t* test was used to evaluate changes in glycogen content and phosphorylated AMPK of lower limb muscles immediately after acute stimulation with the belt electrode. *CONT* unstimulated control group, *ES* group electrical stimulation by belt electrode; *p < 0.05, **p < 0.01.

There is no evidence that B-SES induces simultaneous contraction of lower limb muscles. Thus, we evaluated whether B-SES sufficiently activated the lower extremity muscles and enhanced mitochondrial biosynthesis by quantifying the glycogen content and AMPK phosphorylation after B-SES treatment. Glycogen content decreased in both TA and GAS muscles of the bilateral lower limbs immediately after stimulation (Fig. 1a). AMPK phosphorylation also increased in both TA and GAS of the bilateral lower limbs immediately after acute stimulation (Fig. 1b). These results suggest that B-SES simultaneously induces contractions and enhances mitochondrial biosynthesis in the anterior and posterior surface muscles of the bilateral lower limbs.

### Attenuation of denervation-induced muscle atrophy by chronic belt electrode skeletal muscle electrical stimulation

**Figure 2:**
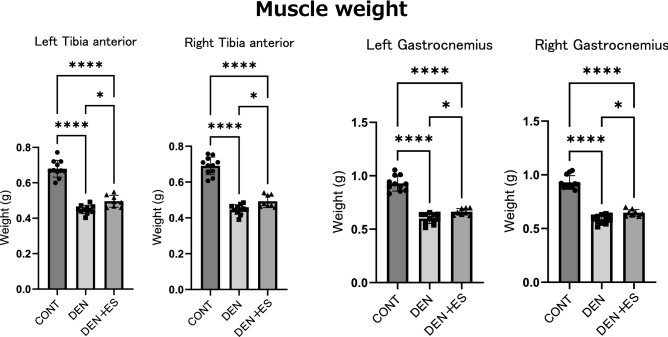
Muscle wet weight of left and right GAS and TA after chronic belt electrode stimulation. Mean ± SD. One-way ANOVA test was used to evaluate changes in the wet weight of the lower limb muscles 24 h after chronic stimulation with a belt electrode. *CONT* unstimulated control group, *DEN* denervation muscle atrophy induced group, *DEN + ES* denervation muscle atrophy induced + by belt electrode electrical stimulation group; *p < 0.05, **p < 0.01, ***p < 0.001, ****p < 0.0001.

**Figure 3:**
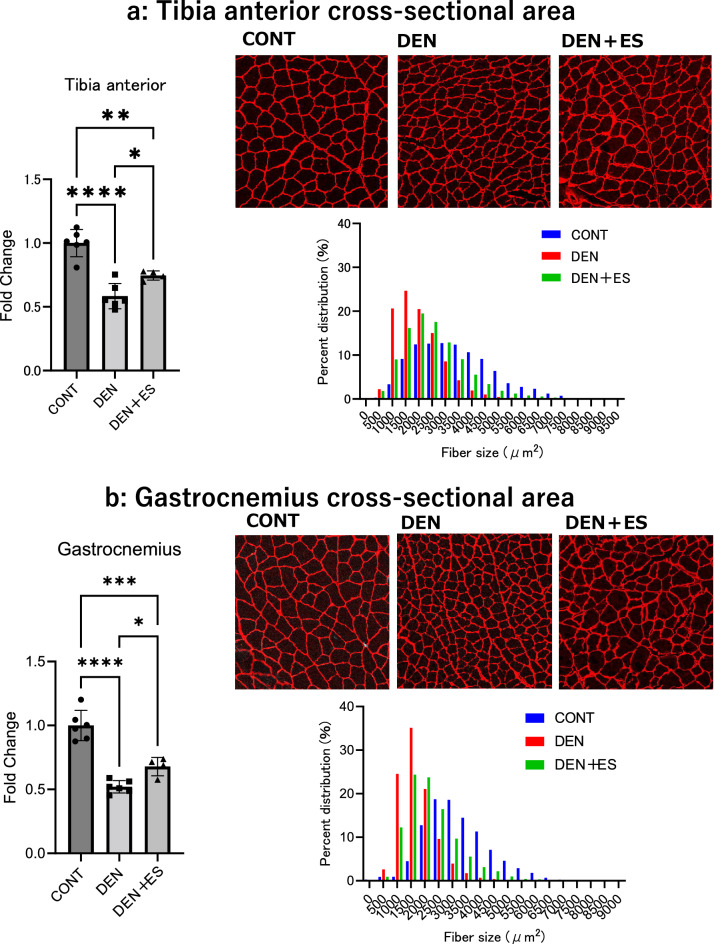
Muscle fiber cross-sectional area (CSA) of TA (**a**) and GAS (**b**) after belt electrode chronic stimulation. Mean ± SD. One-way ANOVA test was used to evaluate changes in the muscle fiber CSA of the lower limb muscles 24 h after chronic stimulation with belt electrodes. CONT, unstimulated control group; DEN, denervation muscle atrophy induced by DEN + ES, denervation muscle atrophy induced + electrical stimulation by belt electrode group; *p < 0.05, **p < 0.01, ***p < 0.001, ****p < 0.0001.

In chronic stimulation, we first evaluated the effect of B-SES on sciatic neurectomy-induced muscle atrophy based on muscle weight and cross-sectional area (CSA). Although the muscle weights of the TA and GAS in the denervation-induced atrophy (DEN) and DEN + electrical stimulation (ES) groups were significantly lower than those in the control group, the muscle weights of the DEN + ES group were significantly higher than those in the DEN group (Fig. 2) (Body weight, CONT: 394 ± 13 g, DEN: 361 ± 23 g, DEN + ES: 369 ± 28 g, mean ± SD). The effect sizes are presented in Table 1. The effect sizes for DEN vs DEN + belt ES were above 0.6, suggesting that the differences had sufficient power. Furthermore, CSA analysis showed that the CSAs of DEN + ES were significantly higher than those of DEN alone (Fig. 3). These results provide clear evidence that B-SES attenuates atrophy in multiple muscle groups.

**Table 1:** Effect size of muscle weight (DEN vs DEN + belt ES).

Muscle	Effect size (r)
Left MG	0.631
Right MG	0.652
Left TA	0.652
Right TA	0.613

### Muscle proteolytic signals after chronic belt electrode skeletal muscle electrical stimulation

**Figure 4:**
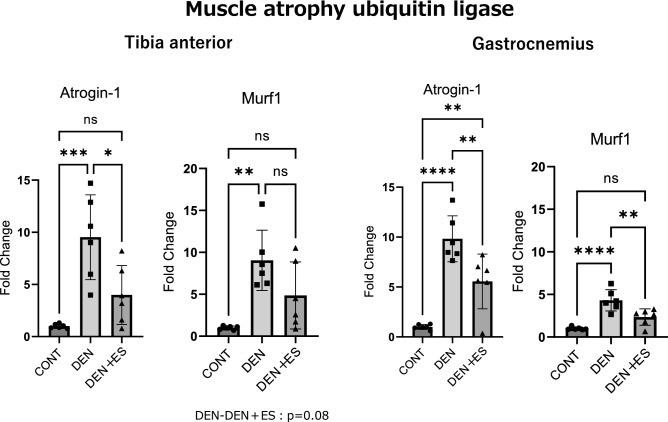
Muscle proteolytic signals of TA and GAS after belt electrode chronic stimulation. Mean ± SD. One-way ANOVA test was used to evaluate changes in proteolytic signals in the lower limb muscles 24 h after chronic stimulation with the belt electrode. CONT, unstimulated control group; DEN, denervation muscle atrophy induced group; DEN + ES, denervation muscle atrophy induced + electrical stimulation by belt electrode group; *p < 0.05, **p < 0.01, ***p < 0.001, ****p < 0.0001.

Next, the muscle proteolytic signals Atrogin-1 and Murf1 after chronic stimulation were evaluated using RT-PCR. Although the mRNA levels of Atrogin-1 and Murf1 in the DEN and DEN + ES groups were significantly higher than those in the control (CONT) group, the mRNA levels of Atrogin-1 and Murf1 in the DEN + ES group were significantly lower than those in the DEN group, indicating that B-SES suppressed the increase in muscle proteolysis caused by denervation in multiple muscle groups (Fig. 4).

### Mitochondria-related signals and enzyme activities after chronic belt electrode skeletal muscle electrical stimulation

We confirmed that acute stimulation upregulated AMPK phosphorylation, suggesting that mitochondrial biosynthesis was enhanced. In this experiment, we evaluated mitochondria-related signals, mitochondrial content, and enzyme activity.

**Figure 5:**
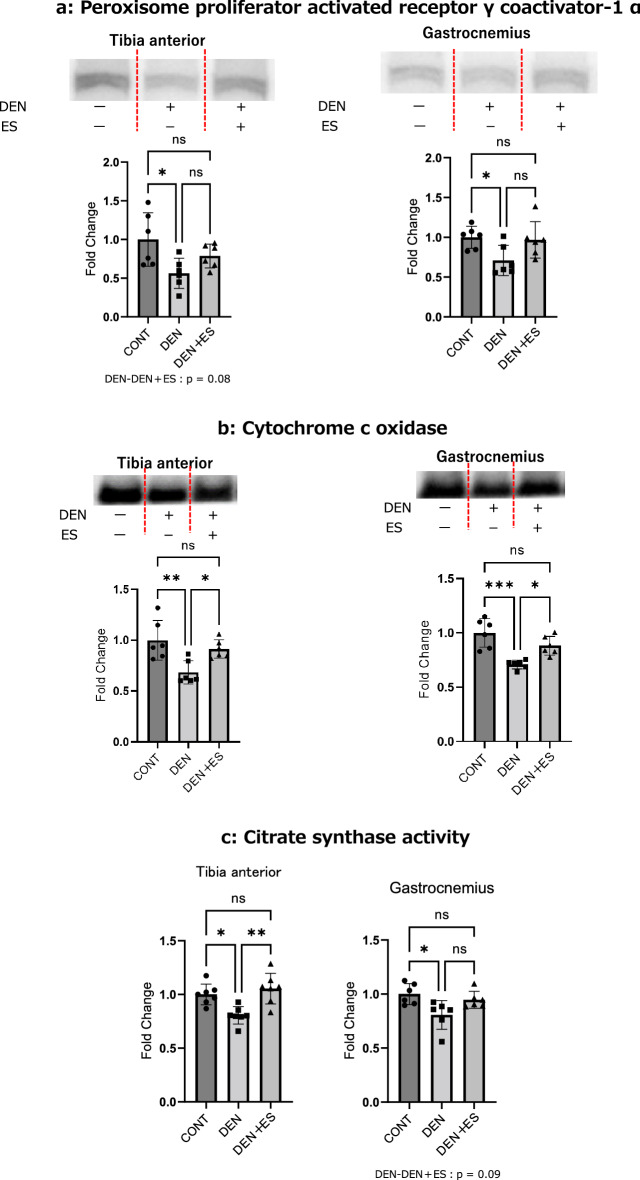
PGC-1α (**a**), COXIV (**b**), and CS activity (**c**) of TA and GAS after chronic belt electrode chronic stimulation. Mean ± SD. One-way ANOVA test was used to evaluate changes in PGC-1α and COXIV levels and CS activity in the lower limb muscles 24 h after chronic stimulation with a belt electrode. *CONT* unstimulated control group, *DEN* denervation muscle atrophy induced group, *DEN + ES* denervation muscle atrophy induced + electrical stimulation with belt electrode group; *p < 0.05, **p < 0.01, ***p < 0.001. All samples were derived from the same experiment and the gels/blots were processed in parallel. All blots are listed in the Supplementary Information.

The expression of PGC-1α, a powerful regulator of mitochondrial biosynthesis, was significantly lower in the DEN group than in the CONT group, but there was no significant difference between the values in the DEN and DEN + ES groups. Regardless, its expression in DEN + ES was higher (p < 0.1) than in DEN (Fig. 5a), suggesting that the decrease in PGC-1α expression due to denervation was suppressed by electrical stimulation.

COXIV protein levels (Fig. 5b) and CS activity (Fig. 5c), which reflect the number of mitochondria, were significantly decreased by denervation in both TA and GAS groups. ES treatment showed that the mitochondrial content was significantly higher in the DEN + ES group than in the DEN group, suggesting that the decrease in mitochondrial content and CS activity due to denervation was suppressed by B-SES.

### Skeletal muscle electrical stimulation induced by pad-type electrode failed to rescue muscle atrophy

Chronic stimulation with belt-type electrodes attached to the bilateral ankle joints successfully suppressed atrophy in both the TA and GAS muscles. To examine whether the form of the electrode influenced the effect of electrical stimulation, a pad-type electrode was applied to both ankle joints with the same amount of electrical current.

**Figure 6:**
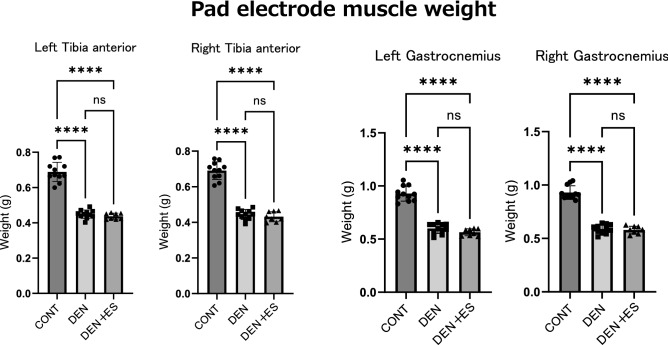
Muscle wet weight of left and right TA, GAS after pad electrode chronic stimulation. Mean ± SD. One-way ANOVA test was used to evaluate changes in the wet weight of the lower limb muscles 24 h after chronic stimulation with pad electrodes. *CONT* unstimulated control group, *DEN* denervation muscle atrophy induced group, *DEN + ES* denervation muscle atrophy induced + belt electrode electrical stimulation group; ****p < 0.0001.

Unlike chronic stimulation with the belt electrode, there was no difference in muscle weight between the DEN and DEN + ES groups when pad-type electrodes were used for skeletal muscle stimulation (Fig. 6) (Body weight, CONT: 394 ± 13 g, DEN: 361 ± 23 g, DEN + ES: 358 ± 15 g, mean ± SD). This result suggests that the belt electrode is effective in suppressing muscle atrophy regardless of the same electrical current.

## Discussion

In this study, we developed a rodent B-SES model and confirmed an acute increase in glucose consumption and AMPK phosphorylation in the TA and GAS of both legs. We also confirmed that B-SES suppressed denervation-induced muscle atrophy in multiple muscle groups after 7-day chronic treatment. A decrease in mitochondrial biosynthesis, quantity, enzyme activity, and muscle proteolytic signals was induced by denervation; however, B-SES treatment rescued these detrimental changes. We found that pad electrodes with the same electrode positions and electrical densities had little effect on skeletal muscle atrophy. In this section, we discuss whether low-frequency B-SES induces activation in multiple skeletal muscles, prevents denervation-induced muscle atrophy, and ameliorates mitochondrial damage.

B-SES has been applied in clinical situations and is reportedly useful in maintaining muscle structure and function in patients^10–12^. In this study, we developed a rodent model of B-SES and evaluated the activation of multiple skeletal muscles. Intramuscular glycogen content is reduced by exercise or EMS-induced skeletal muscle contraction^13^. Additionally, AMPK phosphorylation is induced by energy expenditure during skeletal muscle contraction, which enhances mitochondrial biosynthesis^14,15^. We found that B-SES induced a rapid decrease in intramuscular glycogen content and AMPK phosphorylation in both the anterior and posterior leg muscles (TA and GAS) compared to the control muscles. Glycogen consumption is frequently used as a direct indicator of energy consumption^5,16^. AMPK is an energy sensor, and its phosphorylation is activated when ATP consumption is high, such as during aerobic exercise^17^. Twitch contractions induced by low-frequency electrical stimulation are regarded as aerobic exercise mimics that induce AMPK phosphorylation^15^. Taken together, low-frequency B-SES simultaneously activates multiple muscle groups between the bilateral ankles. Particularly, AMPK activation induces mitophagy and mitochondrial biosynthesis to maintain healthy and functional mitochondria in the cell^14^, which should have a beneficial effect on the maintenance of mitochondrial quantity and function.

In clinical situations, B-SES is used to prevent muscle atrophy in cases of disuse^10–12^; however, the molecular mechanisms have not been fully elucidated. To test whether B-SES has preventive effects on muscle atrophy, we investigated the impact of low-frequency B-SES on muscle atrophy caused by denervation because, as mentioned in the introduction of this report, the decline in nerve transmission from motor nerves to skeletal muscles is thought to occur with aging. After 7 days of treatment, denervation induced a significant decrease in muscle weight and CSA in both TA and GAS muscles, as previously reported^7,18,19^. When denervated rats underwent low-frequency B-SES, both muscle weight and muscle fiber CSA in the TA and GAS were significantly higher than those in denervation-treated muscles, suggesting that B-SES suppresses muscle atrophy simultaneously in multiple muscle groups.

It has been postulated that skeletal muscle mass is the balance between muscle protein synthesis and degradation^20,21^. In muscle wasting, the expression of proteolytic molecules is generally induced^22^, whereas protein synthesis is not always downregulated^23^. In a preliminary study, we found that PI3K-AKT-mTOR signaling was enhanced in denervation-treated atrophied muscles. This is consistent with previous studies^24^. This indicates that it is difficult to use mTOR signaling is used to validate the effect on atrophy inhibition. Thus, in this study, we investigated the mRNA levels of the muscle proteolytic molecules Atrogin-1 and Murf1. We found that Atrogin-1 and Murf1 mRNAs were significantly upregulated by denervation. B-SES significantly suppressed the upregulation of Atrogin-1 and Murf1 mRNAs in the GAS. In TA, Atrogin-1 mRNA was significantly suppressed by B-SES, and Murf1 showed a tendency to be suppressed. Overall, B-SES treatment decreased the proteolytic response that was induced by denervation. Thus, we concluded that B-SES treatment is effective in preventing muscle atrophy, partly via the suppression of muscle protein degradation.

The contraction modes are dependent on the frequency of electrical stimulation. Low- and high-frequency stimulation induce twitch and tetanic contractions, respectively^25^. High-frequency EMS is frequently used to mimic strength-type exercises and induce protein synthesis and muscle hypertrophy^26,27^. In contrast, most previous studies investigating the effect of EMS on denervation-induced atrophy used low-frequency EMS^7,18,28^. Although low-frequency EMS induces mitochondrial biosynthesis^15,29^, its effect on mitochondrial content and function in denervated muscles has not been investigated. Thus, we selected low-frequency electrical stimulation and examined its effect on mitochondria.

Mitochondrial deterioration is frequently observed in various types of muscle wasting^2,4,5,14,30^ and in mitochondrial diseases that cause serious muscle atrophy^31^. Previous reports have clearly shown that in denervated muscles, mitochondrial content and function are severely damaged^2,6^. In this study, we confirmed that the expression of PGC-1α, which contributes to mitochondrial biosynthesis, is reduced by denervation. Cytochrome c content and CS activity also revealed that denervation reduced the mitochondrial content and activity. These results indicated that denervation-induced muscle wasting is accompanied by mitochondrial deterioration. Chronic B-SES treatment rescued reduced PGC-1α expression, COXIV content, and CS activity, suggesting that B-SES treatment has beneficial effects on the muscle mitochondria. As previously discussed, B-SES has been used clinically to prevent skeletal muscle mass loss^10–12^. However, the molecular mechanism of action has not yet been clarified. The results of this study indicate that two-pole low-frequency B-SES simultaneously suppresses denervation-induced muscle atrophy in multiple muscle groups in both legs. This effect was attributed to the suppression of decreased mitochondrial biosynthesis and enzyme activity, which diminished enhanced muscle proteolytic signaling. We used low-frequency EMS, which is a powerful inducer of mitochondrial biosynthesis; however, the effect of high-frequency EMS may be based on different molecular mechanisms, such as mTORC1 signaling and ribosome biosynthesis^26^.

In this study, we found that multiple skeletal muscle groups were simultaneously activated by belt electrodes. We examined whether the pad electrode could suppress muscle atrophy in multiple muscle groups in both legs by attaching it to the posterior portion of both ankle joints, in the same manner as the belt electrode. However, there was no difference in muscle weight between the DEN and DEN + ES groups, and no suppression of muscle atrophy was observed with the pad electrode. This suggests that the belt electrode is critical for simultaneously suppressing muscle atrophy in multiple muscle groups in both legs by applying two electrodes to both ankle joints. However, the method of energizing with pad electrodes used in this study is different from the method normally used in humans. This method was compared it with the one used to stimulate multiple muscle groups when energizing with a two-electrode B-SES. Therefore, whether these results can be applied to humans needs to be elucidated by future studies. Moreover, body length, muscle size, and frequency response may differ between rats and humans, and therefore, further investigation of energizing conditions is necessary when applying this method to humans.

Unlike pad electrodes, which are applied locally to one part of the lower limb, belt-shaped electrodes are tightly attached around the lower limb. This method of wearing a belt electrode allows electricity to flow tubularly throughout the lower limb, facilitating the activation of multiple muscle groups and enabling a more efficient electrical stimulation. Pad electrodes are frequently used in both basic and clinical situations because they are commercially available and easy to use. We used pad electrodes frequently and observed that electrical stimulation with pad electrodes is effective in inducing muscle hypertrophy^32^ and preventing muscle atrophy^33^. On the other hand, we previously confirmed that pad electrodes accidentally stimulate muscles other than the target muscle group, even when two electrodes were put on one muscle group, rat gastrocnemius^34^. This suggests that a multipath electrical current might exist even when stimulating only one muscle group. These results indicate that pad electrodes are easy to use, but attention should be paid to their application. In this experiment, we designed belt electrodes to stimulate multiple muscle groups; however, it has not been confirmed whether belt electrodes activate multiple muscle groups. This study showed that the anterior and posterior muscle groups of the right and left legs were equally activated. This information will help in preventing muscle wasting related to aging and diseases because atrophy occurs in whole-body skeletal muscles.

In this study, the current per area of the pad and belt electrodes were identical. The current values for each electrode differed because of differences in the electrode area. In other words, the belt type is characterized by an increase in the electrode area and a decrease in the current density. Thus, a higher total electrical current can be applied when the belt electrode is used. In clinical settings, a lower electrical current is recommended because of the reduction in sensation (^35^, IEC 60601-2-10). This is another practical advantage of the belt electrode even at the same current intensity.

Finally, this study showed that low-frequency two-electrode B-SES simultaneously activates multiple muscle groups in both legs and suppresses muscle atrophy by suppressing or maintaining mitochondrial content, reducing enzyme activity, and suppressing increased muscle proteolysis. ATP production and enzyme activity by mitochondria in healthy humans increased after 6 weeks of training but decreased after 3 weeks of training suspension^36^, suggesting that continuous training is necessary for mitochondrial content to prevent muscle atrophy and extend the healthy lifespan. However, there are cases in which voluntary and continuous training is difficult, such as after surgery or because of advanced age. EMS is used for rehabilitation because it causes involuntary muscle contraction and can mimic voluntary and continuous exercises under such conditions. The results obtained in this study suggest that the use of a two-electrode B-SES may provide more efficient EMS by activating a wide range of muscle groups, thereby reducing muscle atrophy.

## Methods

### Ethical approval

All experiments were approved by the Animal Experimentation Committee of the Japan Sport Sciences University. All experiments complied with the policies and regulations of the “Basic Guidelines for the Appropriate Conduct of Animal Experiments and Related Activities in Academic Research Institutions” issued by the Ministry of Education, Culture, Sports, Science, and Technology of Japan. The study was conducted according to the ARRIVE guidelines.

### Animals

10-week-old male Sprague–Dawley rats were purchased from CLEA, Japan. The average body weight of all animals was 330 g ± 17 g (mean ± SD). All rats were acclimated by rearing them in the above environment for one week before the experiment. This study involved four different experiments, including acute and chronic belt electrode experiments and acute and chronic pad electrode experiments. All animals were randomly assigned as follows: (1) acute belt experiment, N = 12; (2) chronic belt experiment, N = 30; (3) chronic pad experiment, N = 30. Rats were kept in cages with a 12 h/12 h light/dark cycle (dark time 18:00‒06:00) at 23 °C. All the rats received a standard solid diet (CE-7; CLEA Japan, Tokyo, Japan) and water ad libitum.

### Belt electrode electrical stimulation for acute and chronic belt electrode studies

**Figure 7:**
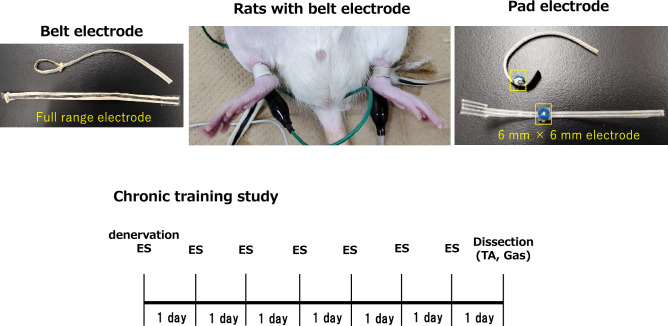
(**a**) belt electrode and (**b**) experimental design. (**a**) Rat with belt electrode anesthetized in the supine position. Hair on the right and left lower limbs was shaved, and belt electrodes were placed on the right and left ankles impregnated with saline. Pad electrodes were similarly placed on the left and right lower extremities behind the left and right ankles and impregnated with saline solution. (**b**) Study design for acute and chronic response. For acute response, the GAS and TA were removed immediately after 30 min of stimulation. For chronic response, the GAS and TA were removed 24 h after the last stimulation. All samples were obtained from the same experiment, and the gels/blots were processed in parallel.

After overnight fasting, the right and left ankle joints were shaved under isoflurane anesthesia (anesthetic aspiration rate: 250‒300 mL/min, concentration: ~ 2.0‒2.2%). The rats were placed on their backs on a platform, and belt-type electrodes (Homer Ion Corp., Tokyo, Japan) were attached to both ankle joints (Fig. 7).

The method of electrical stimulation was modified from previous studies^7,37^. The muscles of the lower extremity were simultaneously stimulated on both sides using electrical stimulation at 7‒8 Hz with a pulse width of 250 μs, eliciting a continuous twitch for 30 min. Stimulation intensity was set to 1.20 mA.

### Pad electrode electrical stimulation chronic pad electrode studies

After overnight fasting, the right and left ankle joints were shaved under isoflurane anesthesia (anesthetic aspiration rate: 250‒300 mL/min, concentration: ~ 2.0‒2.2%). The rats were placed on their backs on a platform, and 6 × 6 mm pad-type electrodes (Homer Ion Corp.) were attached to the posterior side of both ankle joints (Fig. 7).

The muscles of the lower extremity were simultaneously stimulated on both sides using electrical stimulation at 7‒8 Hz with a pulse width of 250 μs, eliciting a continuous twitch for 30 min. The pad electrode was stimulated at an intensity of 0.27 mA with a unified current per area, considering the area difference with the belt electrode (pad electrode area 28.26 mm^2^/belt electrode area 125 mm^2^) × 1.2 mA = 0.27 mA).

### Acute response to belt electrode electrical stimulation

Acute response analysis was performed for two groups: control (CONT) and electrical stimulation (ES).

Under isoflurane anesthesia (anesthetic aspiration rate: 250–300 mL/min, concentration: ~ 2.0–2.2%), a single stimulation with a belt electrode (30 min × 1 set, Fig. 7) was performed. The TA and GAS muscles were removed immediately after exercise. Glycogen consumption, energy expenditure, and mitochondrial biosynthesis due to muscle contraction induced by phosphorylated AMPK were evaluated (n = 6).

### Chronic response to belt electrode electrical stimulation

The chronic response to belt electrode stimulation was analyzed for three groups: control (CONT), denervation (DEN), and denervation + electrical stimulation (DEN + ES).

Chronic response (1 set of 30 min, morning time, 7 consecutive days of stimulation) was performed under isoflurane anesthesia (anesthetic aspiration rate: 250–300 mL/min, concentration: ~ 2.0–2.2%). The TA and GAS were removed after 24 h of the last stimulation (Fig. 7).

Effects of muscle atrophy by muscle weight (n = 8‒11), muscle fiber CSA (n = 4‒6), muscle proteolytic signals, mitochondrial biosynthesis signals, COX IV protein expression, and mitochondrial enzyme activity (n = 6), indicators of mitochondrial content, were evaluated.

### Chronic response to pad electrode electrical stimulation

Analysis of chronic response to pad electrode electrical stimulation was performed for three groups: control (CONT), denervation (DEN), and denervation + electrical stimulation (DEN + ES).

Chronic response (1 set of 30 min, morning time, 7 consecutive days of stimulation) was performed under isoflurane anesthesia (anesthetic aspiration rate: 250–300 mL/min, concentration: ~ 2.0–2.2%). The TA and GAS were removed after 24 h of the last stimulation (Fig. 7). Effect of muscle atrophy on muscle weight (n = 8‒11), was elucidated.

### Skeletal muscle analysis

The medial TA and GAS muscles were used in all experiments because gastrocnemius and tibialis are located in the anterior and posterior parts of the lower limbs, respectively. The resulting muscles were cut into two pieces for biochemical analysis and muscle fiber CSA measurements.

For CSA measurements, the GAS and TA muscles were embedded in Optimal Cutting Temperature (O.C.T) compound (Sakura Finetek Japan, Tokyo, Japan) and flash frozen in cooled isopentane (166-00615, Fujifilm Wako Pure Chemicals Corporation, Osaka, Japan). Other GAS and TA muscles were rapidly frozen in liquid nitrogen for western blotting and muscle glycogen content analysis. All muscle samples were stored at − 80 °C until analysis.

### Denervation

Because sciatic denervation induces muscle atrophy, surgical denervation was performed^3^. Under isoflurane anesthesia (anesthetic aspiration rate: 250–300 mL/min, concentration: ~ 2.0–2.2%), a small incision was made in the posterior aspect of the right and left hind limbs to expose the sciatic nerve. The sciatic nerve was transected using small surgical scissors. The skin was closed using surgical glue. After denervation, a lack of active locomotion was observed in both paws, from which the nerve was severed.

### Skeletal muscle glycogen content

Frozen muscle (10‒20 mg) was powdered and diluted in a homogenization buffer containing 300 µL of 30% KOH saturated with 100 µL of 1 M Na_2_SO_4_. The samples were boiled at 95 °C and mixed every 10 min for 30 min. After incubation, 480 μL of ethanol was added and centrifuged at 1000×*g* for 5 min at 4 °C. The supernatant was discarded, and the pellet was dried for 1 h.

Next, 200 µL Tris–HCl (pH 6.8) was added, dissolved using a sonicator, and the samples were incubated at 95 °C for 2 h. Finally, glycogen content was determined by measuring the absorbance at 505 nm using a LabAssayTM Glucose Kit (298-65701, Fujifilm Wako Pure Chemicals Corp.). The data obtained were corrected based on the weight of each muscle in the powder form.

### Skeletal muscle fiber cross-sectional area

The experimental methods and CSA analysis were modified from those in previous studies^38–40^. GAS and TA muscle bellies were cut into 10-μm-thick frozen sections using a cryostat (CM-502, Sakura Finetech Japan).

Samples were blocked with 5% goat serum (PCN5000, Thermo Fisher Scientific Japan, Tokyo, Japan) for 1 h and incubated with primary anti-laminin antibody (L8271, 1:1000, Sigma Aldrich Japan, Tokyo, Japan) for 2 h. The primary antibody was diluted with a blocking reagent at room temperature. After incubation, the samples were washed with 0.1 M phosphate buffer (5 min × 3 times) and incubated with Alexa fluor 488 conjugated secondary antibody (1:2000, A-11008, Thermo Fisher Scientific, Waltham, MA, USA) at room temperature for 2 h. After incubation, samples were washed with 0.1 M phosphate buffer (5 min × 3 times) and mounted with fluorescence anti-fading reagent (12593-64, Nacalai Tesque, Kyoto, Japan). Images were captured using a confocal laser microscope (FV-3000; Olympus, Tokyo, Japan) and quantified using MyoVision (University of Kentucky, Kentucky, USA). Twelve images were acquired from four sections per muscle for analysis using approximately 4500 to 8000 fibers per group.

### Protein extraction and western blotting

Western blotting was performed as previously described^41–43^. Muscle samples were homogenized in radioimmunoprecipitation assay (RIPA) buffer (188-02453, Fujifilm Wako Pure Chemicals Corp.) containing a protease and phosphatase inhibitor cocktail (169-26063/167-24381, Fujifilm Wako Pure Chemicals Corp.). The protein concentrations of the samples were determined using the BCA method (297-73101, Fujifilm Wako Pure Chemicals Corp.). Equal amounts (40 µg) of protein were separated by SDS-PAGE (NW04125BOX, Thermo Fisher Scientific) and transferred to a polyvinylidene fluoride (PVDF) membrane (IB24001, Thermo Fisher Scientific). Protein transfer was confirmed by Ponceau S staining (33427.01; SERVA Electrophoresis GmbH, Heidelberg, Germany). Membranes were blocked with a blocking reagent (NYPBR01, Toyobo, Osaka, Japan) for 1 h, and primary antibodies were diluted with the dilution reagent (NKB-101, Toyobo). Used antibodies are shown in Table 2. After incubation, cells were washed with Tris-buffered saline containing 0.01% Tween-20 (TBST; T9142, Takara Bio Inc.). The membrane was then incubated with a secondary antibody (7074, Cell Signaling Technology) diluted with a reagent (NKB-101, Toyobo) for 1 h at room temperature and washed again with TBST. The protein bands were visualized using fluorescent reagents (SuperSignal West Pico Chemiluminescent Substrate; Thermo Fisher Scientific). iBright 1500 (FL1500, Thermo Fisher Scientific) and iBright Analysis Software (Windows, Thermo Fisher Scientific) were used to scan and quantify the blots. The Ponceau S signal intensity was used as a loading control.

**Table 2:** Antibodies used in western blot analysis.

Protein	Supplier	Product no.
P-AMPK	Cell Signaling Technology	2531
AMPK	Cell Signaling Technology	2532
COX IV	Abcam	ab14744
PGC1α	Millipore	16557

### RT-PCR

RT-PCR was performed as previously described^32^. The muscle samples were homogenized using TRIzol reagent (356203, Thermo Fisher Scientific). Chloroform (163-20145, Fujifilm Wako Pure Chemicals Corp.) was added to the homogenized samples, mixed, and allowed to stand for 15 min. Muscle samples were centrifuged at 4 °C and 12,000×*g* for 15 min. After collecting the supernatant, ethanol was added and mixed. Total RNA was extracted using an RNA extraction kit (74106; QIAGEN, Hilden, Germany).

Total RNA concentration was measured using NANODROP ONE (Thermo Fisher Scientific), and 1500 ng of total RNA was extracted using a High-Capacity cDNA RT kit (Applied Biosystems, Foster City, CA, USA). Reverse transcription was performed using cDNA. Real-time PCR was performed using the SYBR gene expression assay (Applied Biosystems) in an optical reaction module with a thermal cycler (CFX96, Bio-Rad, California, USA), using the primers (Table 3).

**Table 3:** Primer sequences used in RT-qPCR analysis.

Target	Forward	Reverse
Atrogin-1	AAGGAGCGCCATGGATACTG	AGCTCCAACAGCCTACTACG
Murf1	GACATCTTCCACGCTGCCAA	TGCCGGTCCATGATCACTTC

### Mitochondrial enzyme activity

The method described by Spinazzi et al.^44^ was used to measure CS activity. The tissues were crushed in homogenate buffer (sucrose/Tris/MgCl/015-21274/207-06275/132-001751, Fujifilm Wako Pure Chemicals Corp.) using a biomasher and further crushed using a sonicator. The tissue was then centrifuged at 500×*g* for 5 min at 4 °C and the supernatant was collected. The protein concentration of the collected supernatant was determined using the BCA method (297-73101, Fujifilm Wako Pure Chemicals Corp.). 5 μL of sample solution was diluted to an equal volume (1 mg/mL) of protein and 50 µL of 1 mM DTNB (043-16403, Fujifilm Wako Pure Chemicals Corp.), 10 µL of 3 mM acetyl CoA (00546-96, Nacalai Tesque, Kyoto, Japan), 9 µL of 10% TritonX-100, 1 µL of 1 M Tris–HCl (pH 8.0), and 20 µL of 10 mM oxaloacetic acid (00546-96, Nacalai Tesque) were added. After mixing, changes in absorbance at 412 nm were immediately measured using a multimode plate reader (Tecan, Menendorf, Switzerland) at 37 °C for 15 min at 5 s intervals. The results were analyzed using SparkControl Magellan 2.2, and CS activity was evaluated by analyzing five consecutive changes in absorbance with the largest slope per minute.

### Statistical analysis

Data are presented as the mean ± SE. For acute stimulation, the muscle drive was evaluated using an unresponsive parametric unpaired *t* test to compare the two groups. For chronic stimulation, changes in muscle wet weight, CSA measurements, muscle proteolytic signals, mitochondria-related signals, and enzyme activity were evaluated using a parametric one-way ANOVA test. Statistical significance was defined as p < 0.05, and statistical evaluation was performed using Graph statistical analysis software. GraphPad Prism (version 8.3.0, GraphPad Software, San Diego, CA, USA) was used for statistical analyses.

## Supplementary Information

Supplementary Figures.

## Data Availability

The data supporting the findings of this study are available from H.U. upon reasonable request.
