# Supplementary material for: Low-frequency electrical stimulation of bilateral hind legs by belt electrodes is effective for preventing denervation-induced atrophies in multiple skeletal muscle groups in rats
Source: Sci Rep. 2022 Dec 8;12:21275. doi: 10.1038/s41598-022-25359-z (PMC9732041; doi:10.1038/s41598-022-25359-z)
Supplement: Supplementary file 1 — Supplementary Figures. [file 41598_2022_25359_MOESM1_ESM.pdf]

Figure 2

b: Phosphorylated AMPK

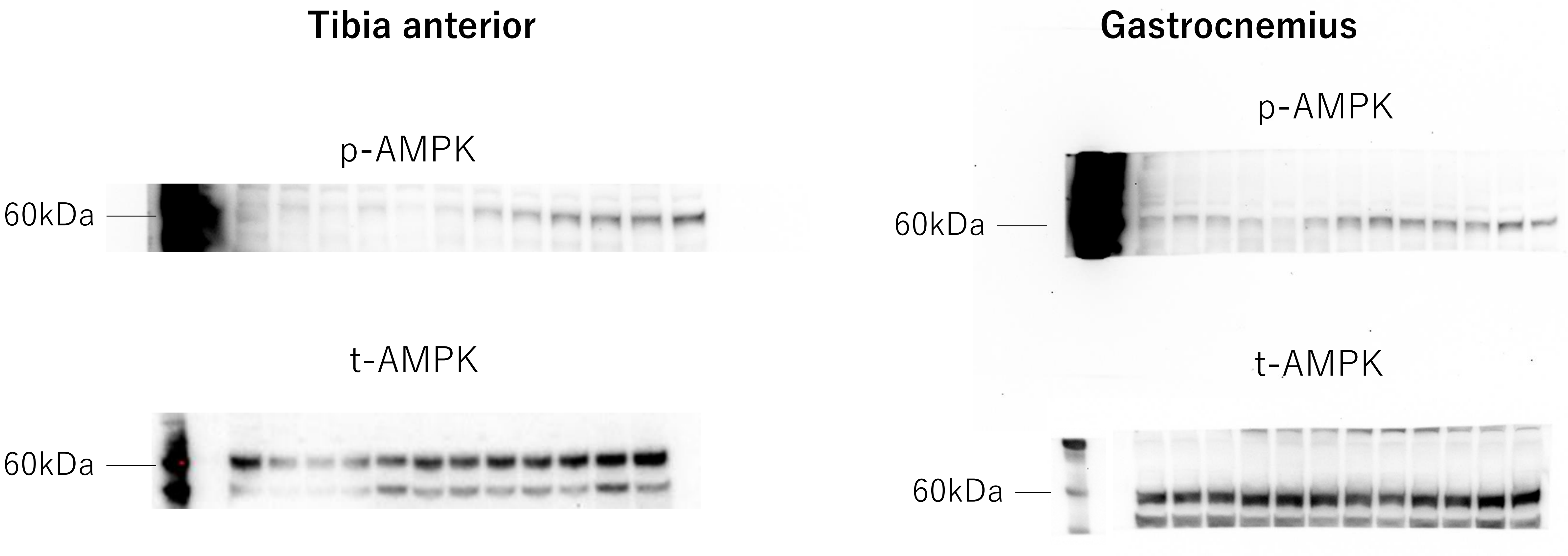

The membrane was cut at the position of the target molecular weight when the antibody reaction.

Figure 6

a: Peroxisome proliferator activated receptor γ coactivator-1 α

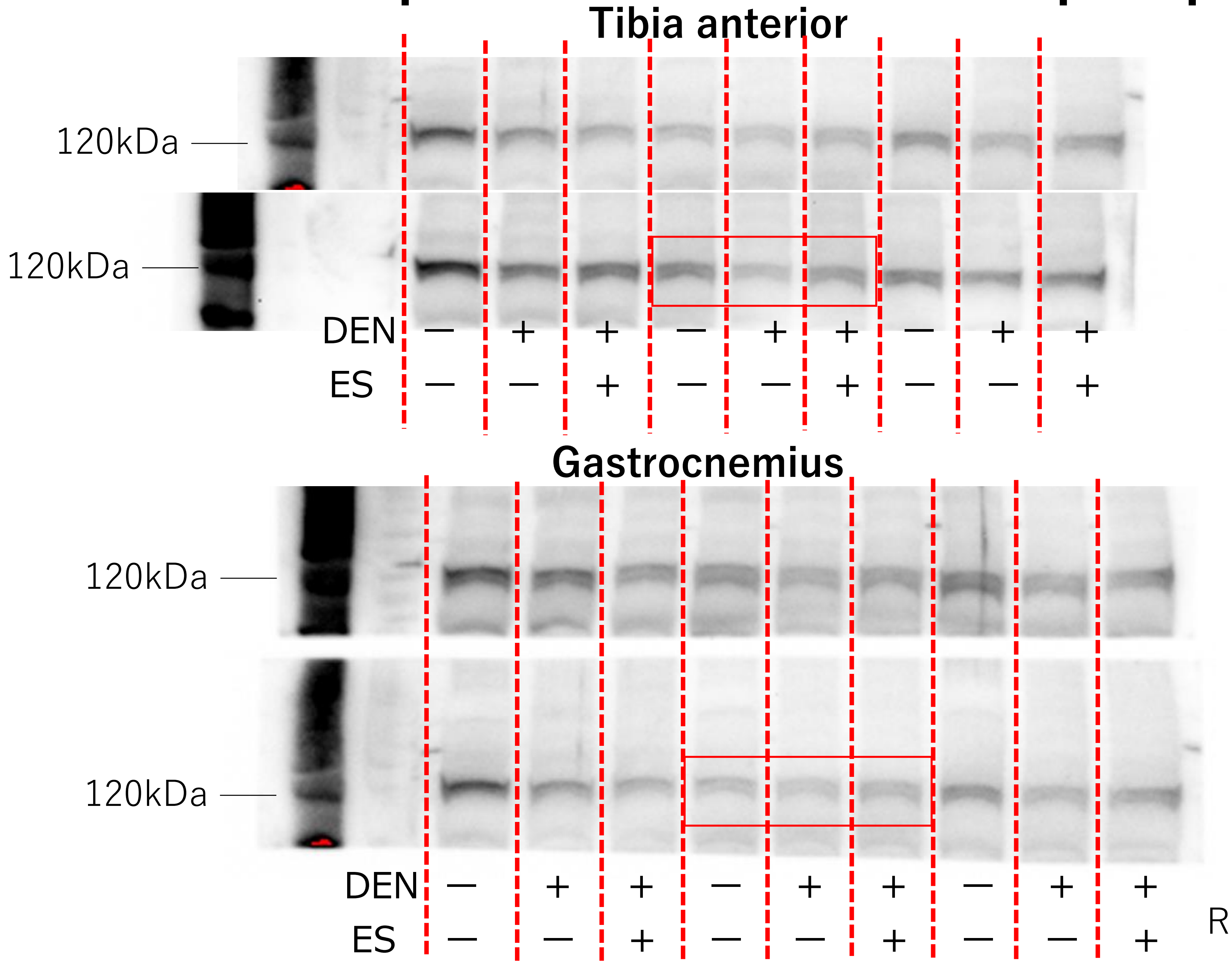

Red frame: used in the figure

The membrane was cut at the position of the target molecular weight and the antibody reaction was performed.

Figure 6

b: Cytochrome c oxidase

Tibia anterior

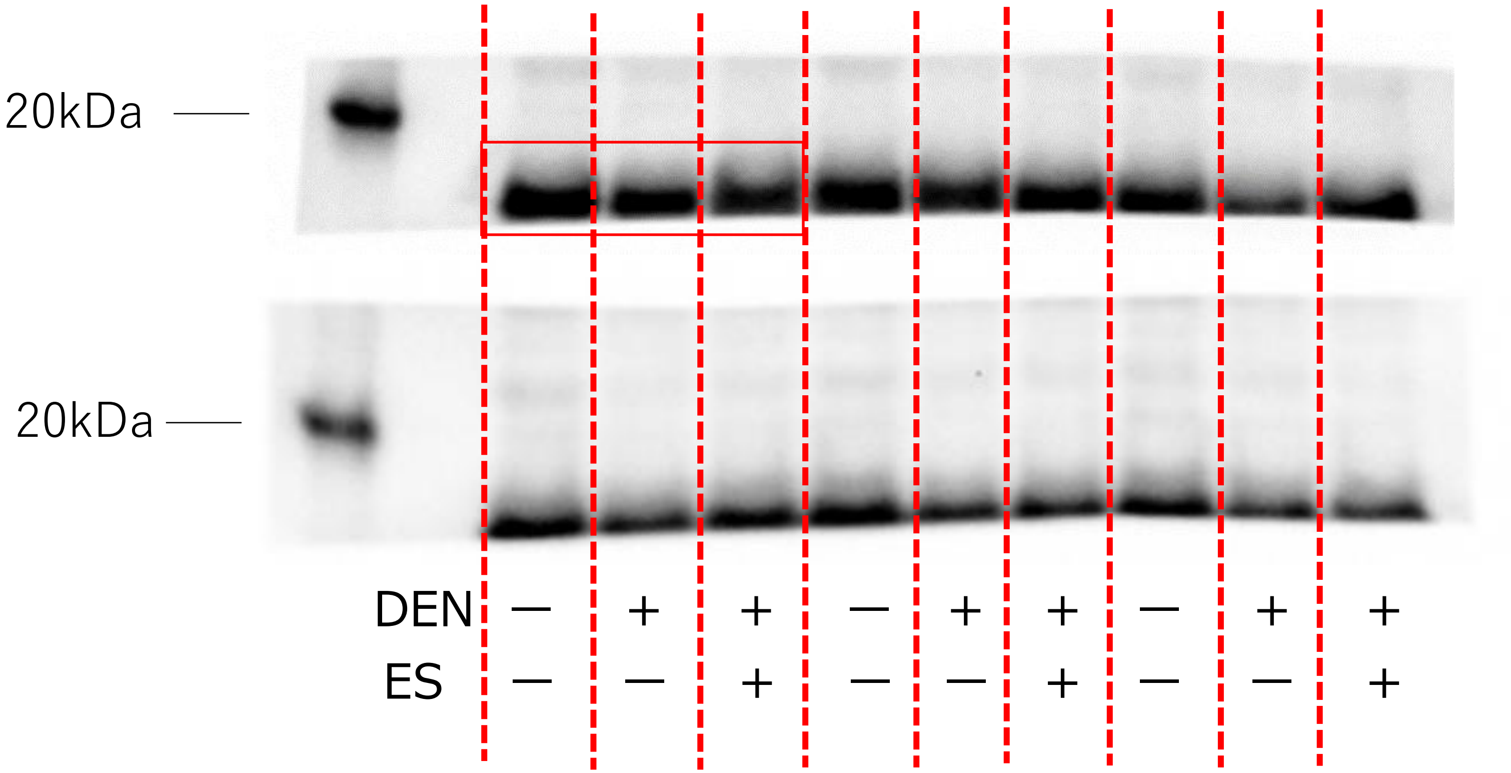

Figure 6

b: Cytochrome c oxidase

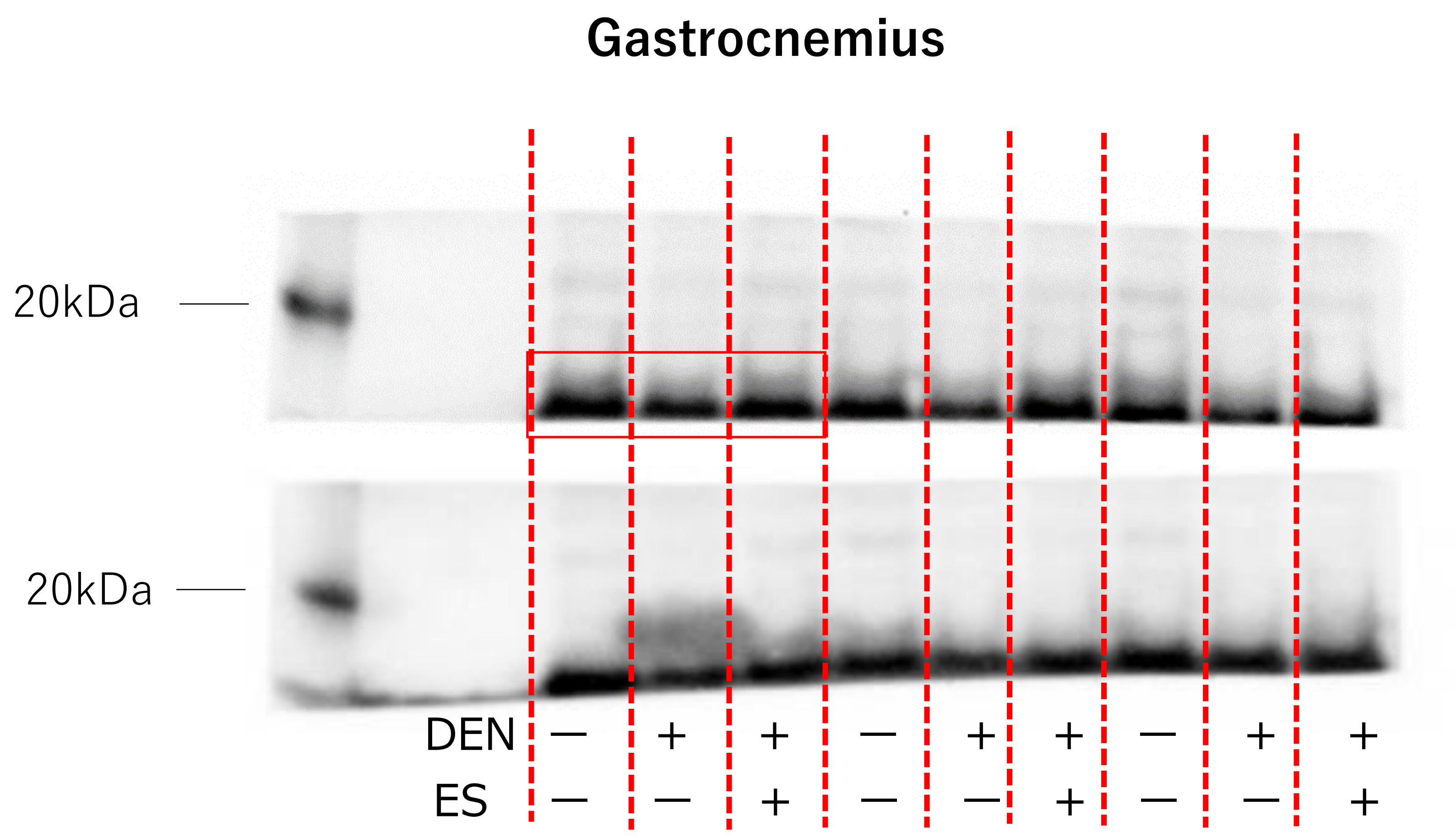

Red frame: used in the figure

The membrane was cut at the position of the target molecular weight when the antibody reaction.
